# Elicitors Derived from Hazel (*Corylus avellana* L.) Cell Suspension Culture Enhance Growth and Paclitaxel Production of *Epicoccum nigrum*

**DOI:** 10.1038/s41598-018-29762-3

**Published:** 2018-08-13

**Authors:** Mina Salehi, Ahmad Moieni, Naser Safaie

**Affiliations:** 1 Plant Breeding and Biotechnology Department, Faculty of Agriculture, Tarbiat Modares University, Tehran, P.O. Box 14115-336, Iran; 2 Plant Pathology Department, Faculty of Agriculture, Tarbiat Modares University, Tehran, P.O. Box 14115-336, Iran

## Abstract

The microbial fermentation is considered as the potential source for large-scale production of paclitaxel. Since co-cultivation/mixed fermentation strategy has been reported as a yield enhancement strategy for paclitaxel production, investigation of fungal endophyte response to plant culture medium, plant cell extract (CE) and medium filtrate (MF) of plant cell suspension culture in terms of growth and paclitaxel production is interesting. In this study, 35 endophytic fungi were isolated from *Taxus baccata and Corylus avellana* grown in Iran. The analysis of high-performance liquid chromatography and mass spectrometry showed that one isolate (YEF_2_) produced paclitaxel. The isolate YEF_2_ was identified as *Epicoccum nigrum* by sequencing of ITS1-5.8S-ITS2 rDNA region and actin gene. YEF_2_ was slow-growing in Murashige and Skoog medium, but the synergistic interaction of gibberellic acid (GA_3_) and CE of *C. avellana* enhanced the growth of YEF_2_. The highest total yield of paclitaxel (314.7 µg/l; 11.5-folds) of *E. nigrum* strain YEF_2_ was obtained by using 28% (v/v) filter sterilized CE of *C. avellana* and 2 µg ml^−1^ GA_3_ that was significantly higher than the control. In this study, the effects of the plant cell extract on growth and paclitaxel production of paclitaxel producing endophytic fungus were studied for the first time.

## Introduction

Paclitaxel, a main impressive chemotherapeutic agent against a wide range of cancers^1^ was originally extracted from the bark of *Taxus brevifolia*, yew native to the North-Western Pacific area and its chemical structure was elucidated in 1971^2^. Paclitaxel stabilizes microtubules to depolymerization, thus arrest the division of actively growing tumor cells at G1 or M phases^3^. Unfortunately, yew trees are slow-growing and large amounts of bark are required for paclitaxel production^4^. Owing to overexploitation, many species are now endangered and on the brink of extinction^5^. Therefore, search for alternative sources of the drug is prompted. In 1993, the first paclitaxel-producing fungus was isolated from the Pacific yew^6^. This initial discovery was followed by the plethora of different endophytic fungi reported producing paclitaxel^7,8^. A microbial fermentation process would be the most favorable means of paclitaxel supply. Microorganisms are fast growing and their genetic manipulation is relatively easy and can be scaled-up to an industrial level. Therefore, microbial fermentation is considered as the potential source for large-scale production of paclitaxel^9^. Low productivity of paclitaxel in endophytic fungi is a drawback for commercial production. Despite the various genera of endophytes capable of producing paclitaxel^9^, there have been no major breakthroughs regarding to commercial production of paclitaxel by fungal fermentation. The problems including the inconsistent production of fungal paclitaxel by repeated sub-culturing on defined artificial media^10^ have raised doubts about the commercial possibility of endophytic fungi as sustainable production platforms. Venugopalan *et al*.^11^ stated that optimization of culture parameter including the exogenous addition of elicitors to *in vitro* cultivation of endophytic fungi improves production of secondary metabolites. It is reported that adding the host plant extracts to the fungal culture was successful in some cases^12^. Host plants affect metabolic processes of the endophytes^13^ and reciprocally transcription of rate-limiting genes in plant paclitaxel biosynthetic pathway is increased by fungal endophytes^14^. Indeed, during the long period of co-evolution, a friendly relationship has been gradually organized between each endophytic fungus and its host plant, so that host plant provides plentiful nourishment for endophytes and fascinates their inhabitation leading to the survival of these endophytes. The endophytes, in turn, synthesize several bioactive compounds for protecting the host plants against biotic and abiotic stresses and boosting their growth^15,16^. In some cases, endophytic fungi have gained the capability of producing identical or similar bioactive compounds as those produced by their host plants. It seems the co-cultivation or the mixed fermentation can mimic the natural habitat of endophytes. Co-cultivation/mixed fermentation strategy has been reported as a yield enhancement strategy for paclitaxel production^17^. It is stated that the addition of *Catharanthus roseus* extract and ethanol in the medium enhanced the camptothecin production in the suspension culture of *Fusarium solani*^18^. It assumes the addition of plant cell extract to suspension culture of paclitaxel-producing endophytic fungi or the co-cultivation of these endophytes with plant cells can provide the required stimulus to the fungal endophyte in the axenic culture for enhanced and sustainable production of paclitaxel.

In addition to *Taxus* spp., hazel (*Corylus avellana*) has also been described as a paclitaxel-producing species through bioprospection among angiosperms^19–21^. The major advantage of producing taxanes through hazel cell suspension culture (CSC) is that hazel is widely available, grows more quickly *in vivo*, and is easier to cultivate *in vitro* than yew^22^.

In the light of importance *in vitro* cultures of *C. avellana* as a promising and cheaper source for paclitaxel production^23^, It seems that the co-cultivation of *C. avellana* cells with paclitaxel-producing endophytic fungus can be promising for enhancing paclitaxel production. Therefore, investigation of fungal endophyte response to plant culture medium, cell extract (CE) and medium filtrate (MF) of *C. avellana* CSC in terms of growth and paclitaxel production is crucial.

The objectives of this study were (a) to isolate endophytic fungi from *Taxus baccata* and *C. avellana* grown in Iran, (b) to screen and identify paclitaxel producing isolates and (c) to investigate the response of paclitaxel-producing endophytic fungus (growth and paclitaxel production) to CE and MF of *C. avellana* CSC as well as GA_3_.

## Results

### Isolation, screening and identification of the paclitaxel-producing endophytic fungus

**Figure 1:**
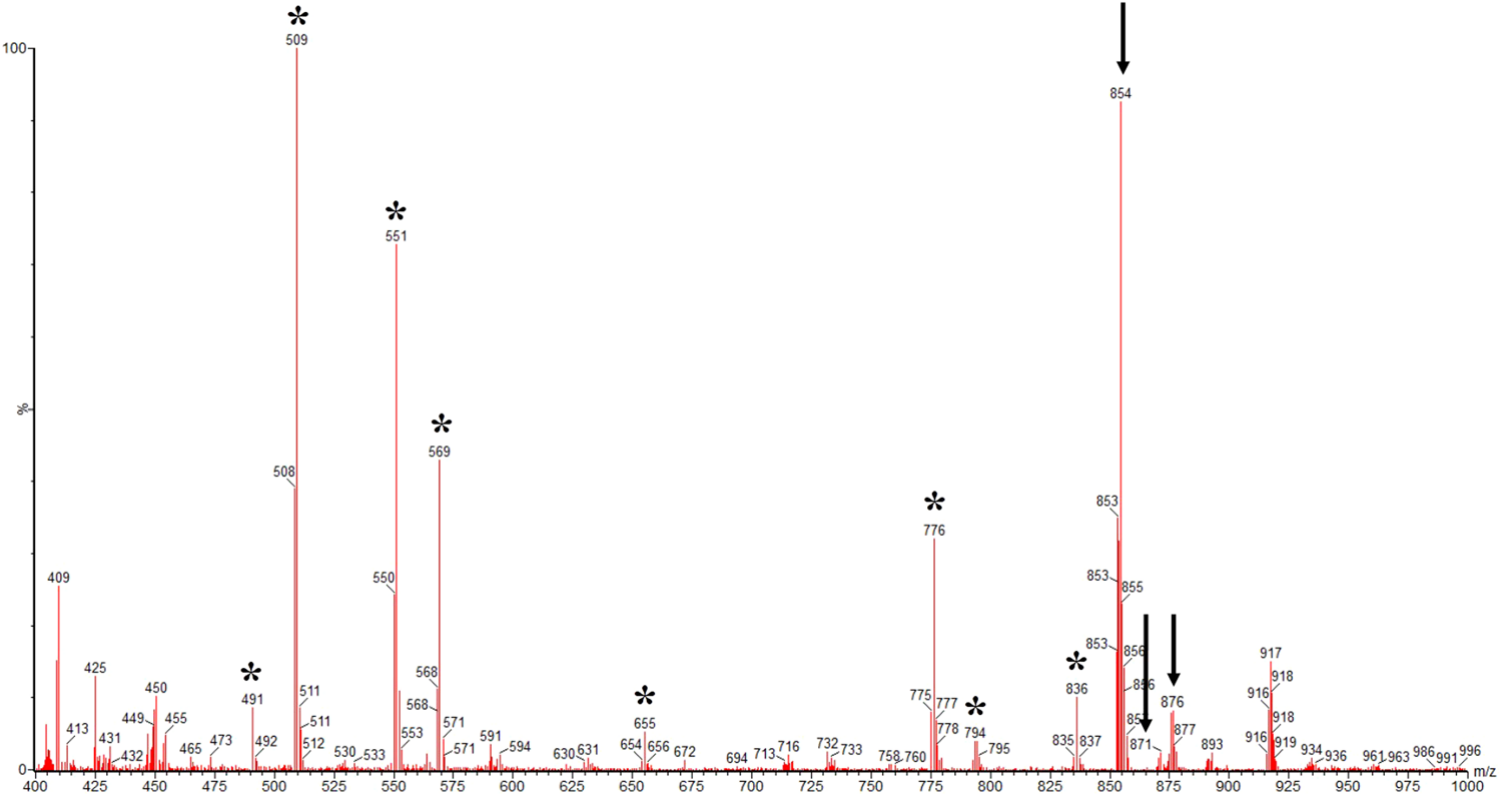
Mass spectrometric analysis of fungal paclitaxel sample of strain YEF_2_. The arrows show an (M + Na^+^) ion with mass-to-charge ratio (m/z) 876, an (M + H^+^) ion with m/z 854 and an (M + NH4^+^) ion with m/z 871 and asterisks indicate fragments ions of mass spectroscopy of paclitaxel that is necessary for identifying paclitaxel.

A total of 35 isolates was separated from *T. baccata* and *C. avellana*. Paclitaxel was extracted from culture filtrates and mycelia of fungi and then analyzed by HPLC. The HPLC analysis of these isolates showed that the peak positions of two strain (YEF_2_ and HEF_12_) were identical to that of standard paclitaxel (retention time = 4.59 ± 0.05 min) (Fig. S1), indicating these fungal isolates may produce paclitaxel. Analysis of paclitaxel production of these two isolates in six passages showed that only production of strain YEF_2_ was stable. This strain was isolated from *T. baccata* bud. Further confirmation for the identity of the paclitaxel was gained by LC-MS/MS. Figure 1 shows representative mass spectra of paclitaxel from strain YEF_2_. The selected ions for the paclitaxel standard are an (M + Na^+^) ion with mass-to-charge ratio (m/z) 876, an (M + H^+^) ion with m/z 854 and an (M + NH_4_^+^) ion with m/z 871^24,25^. The peaks of fungal paclitaxel exhibited m/z ratios corresponding to these molecular ions that confirm this fungal strain can generate paclitaxel. The asterisks on the spectra (Fig. 1) indicate fragments ions which were most helpful for identifying the paclitaxel^20,25^.

By analysis of the sequences of ITS1-5.8S-ITS2 Region and actin gene, this paclitaxel producing strain was identified as *Epicoccum nigrum* (YEF_2_). Endophytic fungal strain YEF_2_ was deposited in Iranian Fungal Culture Collection (WDCM939) under the accession number IRAN 2950C. The partial sequences of the ITS rDNA and actin gene obtained from strain YEF_2_ were deposited in GenBank (NCBI) under the accession numbers MF371418 and MF381043, respectively.

It was very attractive to know if elicitors derived from cell suspension of the non-host plant, *C. avellana*, affect growth and paclitaxel production of strain YEF_2_.

### Synergistic interaction of gibberellic acid and CE of *C. avellana* CSC enhanced the growth of *E. nigrum*

*E. nigrum* is slow-growing in MS medium supplemented with GA_3_ (Fig. S2c). The addition of *C. avallana* CE to MS medium at the time of culture did not increase the growth of *E. nigrum* (Fig. S2b), whereas this strain was relatively fast-growing in MS supplemented with GA_3_ and CE of *C. avallana* (Fig. S2a). The best GA_3_ concentration for *C. avellana* cell growth was shown to be 2 µg/ml (data not shown). So, in all next experiments, 2 µg/ml GA_3_ was added to the medium at the time of culture. At the first, three concentrations (3%, 5% and 7% (v/v)) of *C. avallana* CE were added to culture medium and a slight increase in growth was obtained by adding 3% and 5% CE of *C. avellana* (Fig. S3). Therefore, three concentrations (7%, 14%, and 28%) were selected for next experiment.

### The CE and MF of *C. avellana* CSC enhanced growth and total yield of paclitaxel of *E. nigrum* strain YEF_2_

**Figure 2:**
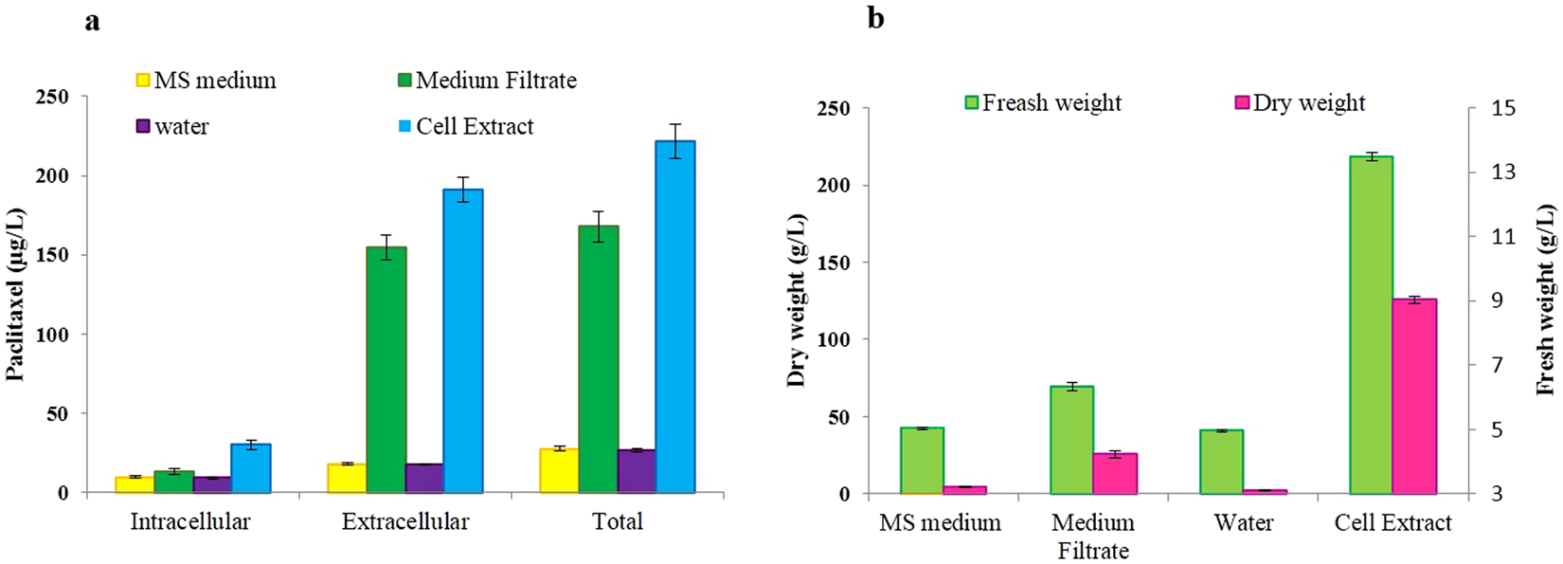
Influence of cell extract (CE) and medium filtrate (MF) of *Corylus avellana* cell suspension culture on paclitaxel production (**a**) and growth (**b**) of *Epicoccum nigrum* strain YEF_2_ grown in MS medium supplemented with 0.2 mg/L BAP, 2 mg/L GA_3_ and 2 mg/L 2,4-D. The controls for CE and MF Were water and MS medium supplemented with 0.2 mg/L BAP, 2 mg/L GA_3_ and 2 mg/L 2,4-D, respectively. Average values are given, error bars are represented by vertical lines.

**Figure 3:**
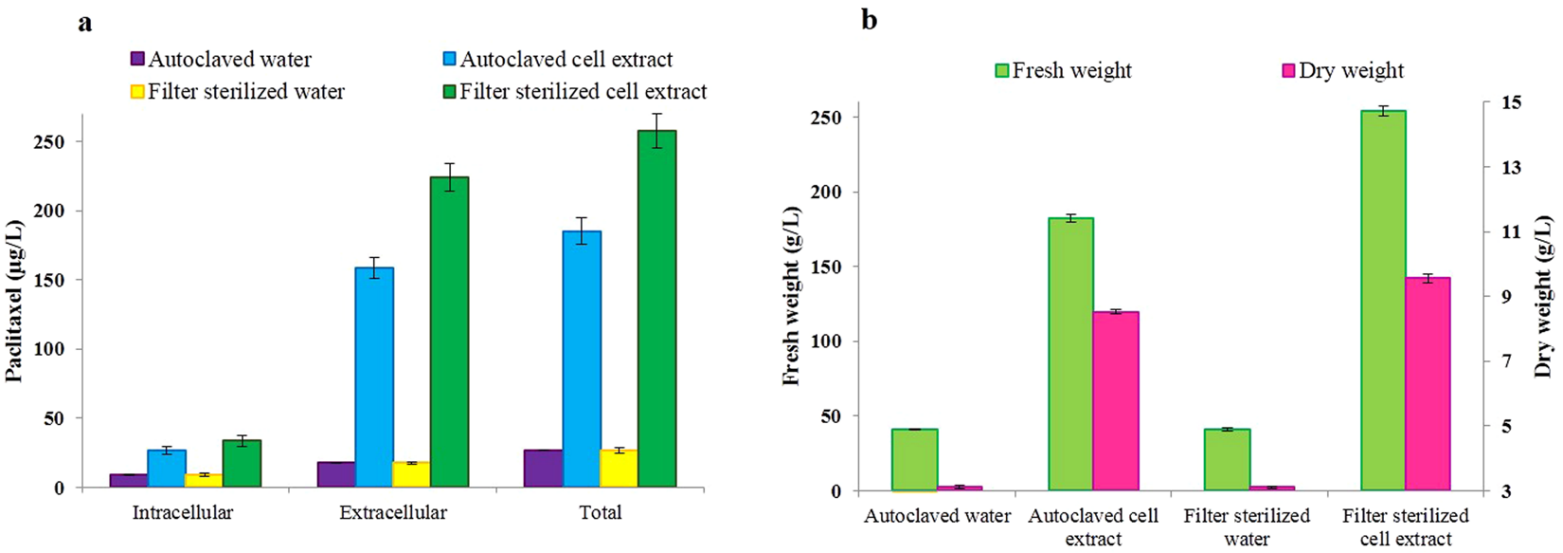
Influence of autoclaved cell extract (ACE) and filter sterilized cell extract (FCE) of *Corylus avellana* cell suspension culture on paclitaxel production (**a**) and growth (**b**) of *Epicoccum nigrum* strain YEF_2_ grown in MS medium supplemented with 0.2 mg/L BAP, 2 mg/L GA_3_ and 2 mg/L 2,4-D. The controls for ACE and FCE were autoclaved water and filter sterilized water, respectively. Average values are given, error bars are represented by vertical lines.

**Figure 4:**
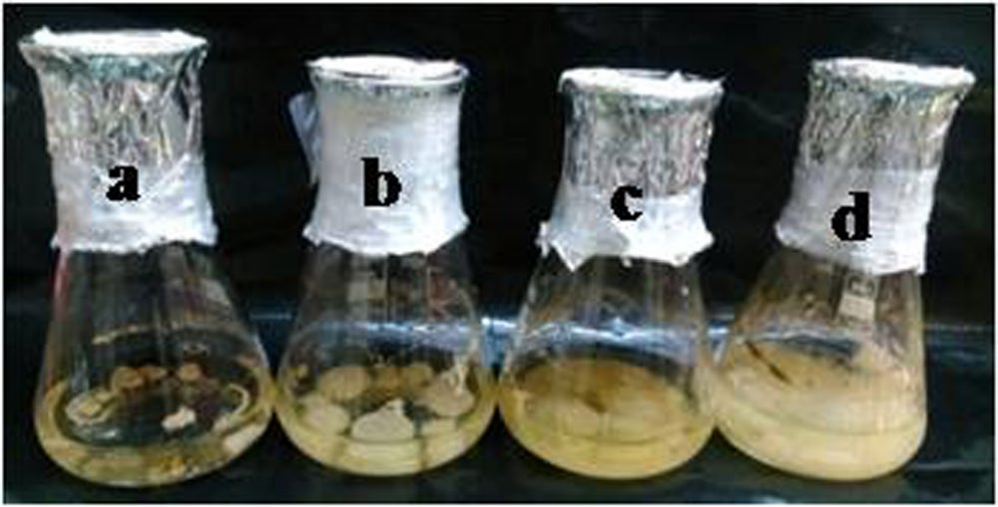
*Epicoccum nigrum* strain YEF_2_ grown in MS medium supplemented with 0.2 mg/L BAP, 2 mg/L GA_3_, 2 mg/L 2,4-D and different concentration of filter sterilized cell extract of *Corylus avellana* cell suspension culture (a to d, 0%, 7%, 14% and 28% (v/v), respectively).

**Figure 5:**
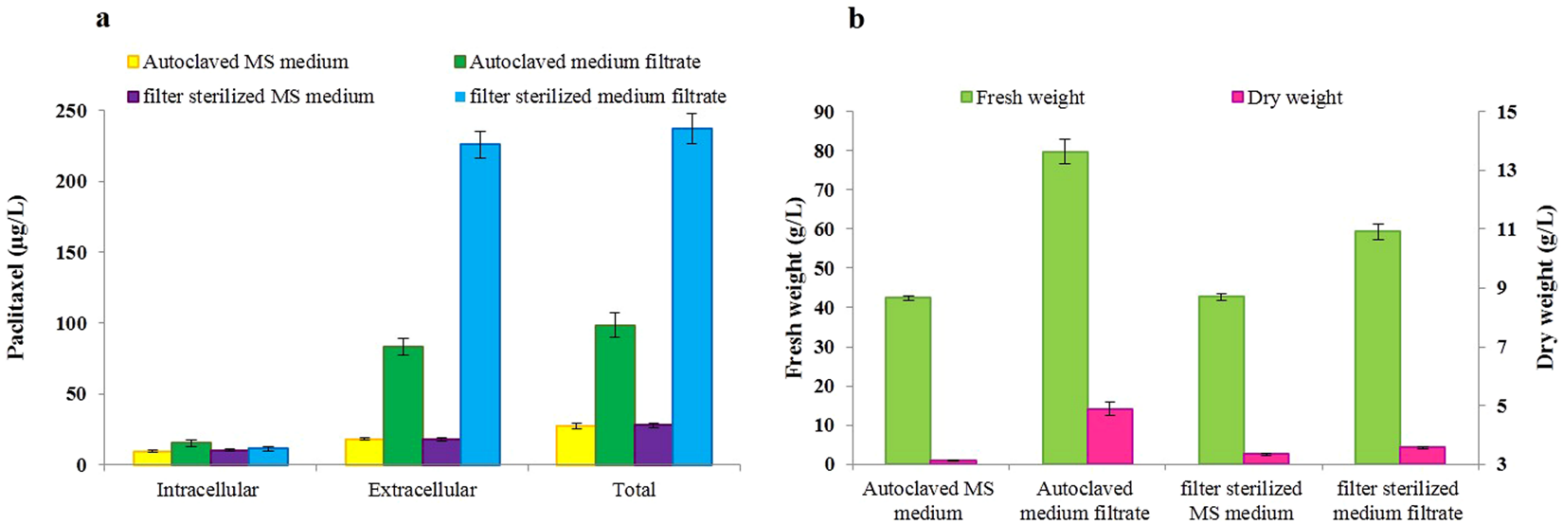
Influence of autoclaved medium filtrate (AMF) and filter sterilized medium filtrate (FMF) of *Corylus avellana* cell suspension culture on paclitaxel production (**a**) and growth (**b**) of *E. nigrum* grown in MS medium supplemented with 0.2 mg/L BAP, 2 mg/L GA_3_, 2 mg/L 2,4-D. The controls for AMF and FMF Were autoclaved MS medium and filter sterilized MS medium (supplemented with 0.2 mg/L BAP, 2 mg/L GA_3_ and 2 mg/L 2,4-D), respectively. Average values are given, error bars are represented by vertical lines.

Paclitaxel in culture medium containing the elicitors derived from *C. avellana* CSC with no inoculums was analyzed (Table S2) and paclitaxel in all treatments was modified based on paclitaxel of the culture medium containing the elicitors with no inoculums. The results of induction of growth and paclitaxel production in *E. nigrum* using CE and MF of *C. avellana* CSC showed that the fresh weight (FW), dry weight (DW), intracellular paclitaxel (µg/l), extracellular paclitaxel and total paclitaxel of the fungus significantly affected by CE and MF of *C. avellana* CSC. The main effects of treatments and their interactions (reciprocal and trilateral effects) on mentioned traits were highly significant. Considering the treatments individually, CE was performed better as plant elicitor in terms of FW (218.1 g/l) (5.3- folds), DW (9 g/l) (2.9- folds), intracellular (30.1 μg/l) (3.3-folds), extracellular (191.3 μg/l) (10.8-folds) and total yield of paclitaxel (221.4 μg/l) (8.2-folds) as compared to the control (Fig. 2). The significant interaction effect of elicitor type (CE or MF) and sterilization method (Filter sterilized or autoclaved) indicated that elicitor type affected measured traits differently depending on used sterilization technique. Because of the significant interaction effect of elicitor type and sterilization technique, the effects of elicitor type were analyzed on each sterilization method. The means comparison indicated that FCE was more effective than ACE for the increase of FW (254. 1 g/l) (6.2-folds), DW (9.6 g/l) (3.1-folds), intracellular (33.8 μg*/*l) (3.6-folds), extracellular (224.0 μg/l) (12.7-folds) and total yield of paclitaxel (257.8 μg/l) (9.6-folds) (Fig. 3). The interaction effect of elicitor type × concentration level indicated that the effect of elicitors was level-dependent (Fig. 4) and the means comparison showed that the highest FW (384. 4 g/l) (9.9-folds), DW (12.6 g/l) (4.1-folds), intracellular (40.3 μg/l) (4.3-folds), extracellular (274.4 *μ*g/l) (15.3-folds) and total yield of paclitaxel (314.7 μg/l) (11.5-folds) of *E. nigrum* strain YEF_2_ were obtained with 28% (v/v) FCE and 2 µg/ml GA_3_ that were significantly higher than the control (supplemented with 2 µg/ml GA_3_ and 28% (v/v) filter sterilized water) with a mean of 38.9 g/l, 3.1 g/l, 9.3 μg/l, 17.9 μg/l and 27.3 μg/l, respectively (Table 1). The results of ANOVA indicated that CE and MF of *C. avellana* CSC increased intracellular (per liter of medium), extracellular and total yield of paclitaxel, whereas no significant increase in intracellular yield of paclitaxel per gram dry weight of mycelia was observed. Total yield of paclitaxel (dependent variable (Y)) was regressed against FW and DW. The regressions of total paclitaxel against FW and DW for FCE, ACE, AMF were positive and significant (Table 2). Additionally, the fitted models for ACE and FCE exhibited high R-Squared. Meanwhile, AMF increased FW (79.7 g/l) (1.9-folds), and DW (4.9 g/l) (1.6-folds) of *E. nigrum* more than FMF (Fig. 5b). However, FMF was more effective for paclitaxel production than the AMF (Fig. 5a). Also, The regressions of total paclitaxel against FW and DW for FMF were not significant (Table 2).

**Table 1:** Effect of various elicitors derived from *Corylus avellana* L. cell suspension culture on growth and paclitaxel production of *Epicoccum nigrum* strain YEF_2_.

Elicitors (% (v/v))	Fresh weight (g/L)	Dry weight (g/L)	Extracellular paclitaxel (μg/L)	Intracellular paclitaxel (μg/g)	Intracellular paclitaxel (μg/L)	Total yield of paclitaxel (μg/L)	Extracellular paclitaxel portion (%)
Control (without elicitor)	43.3 ± 0.0	3.11 ± 0.03	18.2 ± 0.6	2.88 ± 0.17	8.97 ± 0.60	27.2 ± 1.0	67.0 ± 1.4
ACE (7%)	105.6 ± 2.9	6.25 ± 0.04	109.7 ± 6.5	3.05 ± 0.34	19.10 ± 2.22	128.8 ± 8.8	85.3 ± 0.7
ACE (14%)	137.8 ± 2.2	8.07 ± 0.06	146.1 ± 7.0 c	3.33 ± 0.34	26.93 ± 2.86	173.0 ± 8.35	84.5 ± 1.4
ACE (28%)	303.3 ± 1.9	11.22 ± 0.13	219.8 ± 12.3	2.98 ± 0.36	33.57 ± 4.40	253.4 ± 15. 3	86.8 ± 1.2
FCE (7%)	137.8 ± 1.1	7.17 ± 0.15	161.2 ± 10.4	3.79 ± 0.47	27.18 ± 3.37	188.4 ± 9.9	85.5 ± 2.0
FCE (14%)	240.0 ± 3.8	8.86 ± 0.22	236.6 ± 14.4	3.80 ± 0.44	33.79 ± 4.31	270.4 ± 18. 4	87.6 ± 0.89
FCE (28%)	**384.4 **±** 4.8**	**12.64 **±** 0.21**	**274.4 **±** 11.0**	3.18 ± 0.42	**40.30 **±** 5.40**	**314.7 **±** 15.2**	87.3 ± 1.2
AMF (7%)	64.6 ± 2.7	4.05 ± 0.05	76.4 ± 8.2	3.10 ± 0.37	12.60 ± 1.67	89.0 ± 9.32	85.8 ± 1.4
AMF (14%)	72.2 ± 2.2	4.33 ± 0.04	76.9 ± 8.7	3.16 ± 0.36	13.72 ± 1.66	90.6 ± 10.3	84.9 ± 0.7
AMF (28%)	102.2 ± 4.8	6.28 ± 0.59	96.7 ± 3.4	3.08 ± 0.39	19.81 ± 4.22	116.5 ± 7.5	83.3 ± 2.6
FMF (7%)	51.1 ± 1.1	3.06 ± 0.01	224.6 ± 11.9	3.23 ± 0.40	9.90 ± 1.22	234.5 ± 13.07	95.8 ± 0.3
FMF (14%)	60.0 ± 1.9	3.58 ± 0.04	225.3 ± 8.9	3.09 ± 0.43	11.12 ± 1.66	236.4 ± 10.4	95.3 ± 0.5
FMF (28%)	66.7 ± 3.3	4.05 ± 0.03	227.7 ± 9.8	3.22 ± 0.36	13.10 ± 1.59	240.8 ± 11.3	94.6 ± 0.4

Average values ± standard error of mean are given.

Abbreviations are as follows: ACE; Autoclaved cell extract, FCE; Filter sterilized cell extract, AMF; Autoclaved medium filtrate, FMF;Filter sterilized medium filtrate.

**Table 2:** Simple Linear Regression equations and statistics for estimating total yield of paclitaxel (µg/L) of *Epicoccum nigrum* strain YEF_2_ via fresh weight (FW) and dry weight (DW).

Treatment	Equation (Y = total yield of paclitaxel (μgl^−1^))		P-value		R-squared		Correlation Coefficient	
	Against F.W.	Against D.W.	Against F.W.	Against D.W.	Against F.W.	Against D.W.	Against F.W.	Against D.W.
ACE	77.9597 + 0.587827*FW	−30.6762 + 25.3427*DW	0.0001	0.0000	89.2605	93.3489	0.944778	0.966172
FCE	129.25 + 0.505956*FW	51.7963 + 21.5502*DW	0.0007	0.0018	82.5904	77.2331	0.908793	0.878824
AMF	27.6739 + 0.891776*FW	34.5819 + 13.1149*DW	0.0047	0.0094	70.4572	64.1913	0.839388	0.801195
FMF	173.128 + 1.08166*FW	205.097 + 9.0046*DW	0.2078	0.5703	21.5727	4.82169	0.464464	0.219584

ACE; Autoclaved cell extract, FCE; Filter sterilized cell extract, AMF; Autoclaved medium filtrate, FMF; Filter sterilized medium filtrate.

## Discussion

### *E. nigrum as a* Paclitaxel-Producing Endophytic Fungus

*E. nigrum* is a fungal species from the phylum *Ascomycota* and a saprophyte on crop residues^26^. This species can live as an endophyte in different plants^27,28^. In some studies, *Epicoccum* genus was reported as a paclitaxel producing endophytic fungus^10,24,29^. Also, *E. nigrum* is especially known for its biocontrol activity against pathogens^30,31^. *E. nigrum* is yellow initially and then become orange and later brown in late growth stage (Fig. S4). A yellow pigment was extracted from the culture of *E. nigrum* and identified as flavipin (3,4,5- trihydroxy-6-methylphthalaldehyde) which had antifungal activity^32^. Also, the red pigments produced by *E. nigrum* were analyzed and detected to consist of four carotenoids^27,33^. These colorants produced by *E. nigrum* could be used in food, pharmaceutical, textile, and cosmetic industries^34^.

### Synergistic interaction of gibberellic acid and cell extract of *C. avellana* enhanced the growth of *E. nigrum*

Probably the co-cultivation of *E. nigrum* with *C. avellana* cells can enhance the yield of paclitaxel. Therefore, investigating the ability of *E. nigrum* to grow in plant culture media is essential. *C. avellana* cell clone used in this study have cultured in MS medium. MS medium is one of the most commonly used media for plant tissue culture that developed by Murashige and Skoog^35^. The preliminary experiments showed that *E. nigrum* strain YEF_2_ is very slow-growing in MS medium (Fig. S2d). GA_3_ is a hormone found in plants and fungi and reported that it stimulates growth in cellulolytic fungi, i.e., *Chaetomium globosum, Memnoniella echinata* and *Ourvularia lunata*^36^. Therefore, we hypothesized that this hormone may stimulate the growth of *E. nigrum*. El-bahrawy^36^ stated that the fungi differed from each other to the response of GA_3_ effect. It is observed that *E. nigrum* is slow-growing in MS medium supplemented with GA_3_. (Fig. S2c). Since *E. nigrum* strain YEF_2_ is an endophytic fungus of plants and host plant supply plenteous nutrients for the survival of its endophytes in symbiosis relationship, in the next stage we tested the effects CE and MF of *C. avellana* CSC on the growth of *E. nigrum*. The addition of CE of *C. avellana* CSC to MS medium did not increase the growth of *E. nigrum* (Fig. S2b), whereas this strain was relatively fast-growing in MS supplemented with GA_3_ and cell extract of *C. avallana* CSC (Fig. S2a). Indeed, the effects of CE of *C. avallana* CSC and GA_3_ on *E. nigrum* growth were synergistic. Based on our observations, GA_3_ stimulates the growth of *E. nigrum* strain YEF_2_ in the presence of hazel CE. Indeed, GA_3_ was the essential prerequisite for the growth of strain YEF_2_ in the presence of *C. avallana* CE.

### CE and MF of *C. avellana* CSC enhanced growth and total yield of paclitaxel of *E. nigrum* strain YEF_2_

Resident endophytes within plants are steadily interacting with their hosts. It is found that plants would have a substantial influence on *in planta* metabolic processes of the endophytes^13^. For example, the study of the gene cluster expression for lolitrem biogenesis in endophytic *Neotyphodium lolii* resident in *perennial ryegrass* revealed that expression of these genes is high *in planta*, but low *in vitro* fungal cultures^37^. So it seems that fungal paclitaxel production such as fungal growth would be affected by CE and MF of *C. avellana* CSC. Therefore, the effects of CE and MF of *C. avellana* CSC on fungal paclitaxel production were investigated. The CE was performed better as plant elicitor in terms of the growth and paclitaxel production. Indeed, The CE of *C. avellana* supplies plenteous nutrients and enhances the growth of strain YEF_2_. The means comparison indicated that FCE was more effective than ACE for the increase of growth and paclitaxel production (Fig. 3). Autoclaving denatures proteins and degrades amino acids and may decrease the nutritional value of CE which resulted in decreased growth and paclitaxel production. The results of ANOVA indicated that CE and MF of *C. avellana* CSC increased intracellular (per liter of medium), extracellular and total yield of paclitaxel whereas no significant increase in intracellular yield of paclitaxel per gram dry weight of mycelia was observed. Indeed, the part of produced paclitaxel accumulated in mycelia and much of it secreted in the culture medium. Simple linear regression was developed to determined relationship between total yield of paclitaxel and FW and DW. The fitted models for ACE and FCE exhibited high R-Squared which showed these models explain high percent of the variability in total paclitaxel. Also, the high correlation coefficients indicate relatively strong relationship between total paclitaxel and FW and DW (Table 2). It seems that CE (autoclaved and filter sterilized) and AMF of *C. avellana* CSC enhanced the growth of *E. nigrum* strain YEF_2_ and the increase in total paclitaxel production is due to increase biomass rather than a direct effect of the CE and AMF of *C. avellana* CSC. Meanwhile, AMF increased the growth of *E. nigrum* more than FMF (Fig. 5b). However, FMF was more effective for paclitaxel production than the AMF (Fig. 5a). Also, Regressions of total paclitaxel against FW and DW for FMF were not significant (Table 2). It is assumed that there are many precursors of paclitaxel in *C. avellana* CSC and used to produce paclitaxel that these precursors were degraded by autoclaving and lower growth in MS medium supplemented with FMF rather than AMF can be due to increasing paclitaxel production. The inverse relationship between cell growth and paclitaxel accumulation has been reported previously^38,39^. There is one study which has reported the plant cells produced inhibitory substances in the late growth curve of cell suspension that decreased the fungal biomass^17^. The presence of these inhibitors in FMF may be a drawback for the increase of YEF_2_ growth but autoclaving partly degraded these substances. Therefore, MS medium supplemented with AMF increased growth more than FMF. The presence of paclitaxel precursors in plant cell cultures and the decrease of the final contents of these precursors in fungus through fungi fermentation were reported^17^. In this context, further studies may be useful on the initial and final contents of paclitaxel precursors and investigation of converting them to paclitaxel in fungus during fermentation by labeled precursors. Improved growth of *Fusarium mairei* by adding supernatants of yew cell suspension cultures of days 10 and 15 was reported^17^. Also, it is showed that yew needle extract can elicit fungal paclitaxel production. However, no information has been reported regarding the influence of plant cell extract in the culture medium on growth and paclitaxel production of endophytic fungi. We studied for the first time the effects of *C. avellana* CE on growth and paclitaxel production of paclitaxel producing *E. nigrum* strain YEF_2_.

## Conclusion

The major limitation of using endophytic fungi for industrial paclitaxel production are the low and unstable productivity. In this study, the addition of *C. avellana* cell extract to MS medium enhanced growth and paclitaxel production of *E.nigrum* strain YEF_2_. It is thought that addition of hazel cell extract to MS medium in *E. nigrum* culture simulated relatively the chemical environment of its host and resulted in increased growth and paclitaxel production. Since paclitaxel production by endophytic fungi is significantly reduced by repeated sub-culturing on defined artificial media^10^, using plant cell extract in artificial media may be useful. It is essential to examine whether stable enhanced production of paclitaxel can be obtained using plant cell extract in the fungal culture and the co-cultivation of endophytic fungus with plant cells. Also, it is stated that endophytic fungi in artificial media have no access to the specialized microenvironment of the host plant which may lead to silencing of their secondary metabolite genes, and standard culture conditions may not be sufficient to trigger expression of the cryptic biosynthetic gene clusters^40^. Based on achieved results, using plant cell extract in the fungal culture or the co-cultivation of fungi with plant cells can be useful for enhancement of paclitaxel production. Large-scale production and extraction of pharmaceutical compounds from the plants are high-priced and tedious. However, the endophytic fungi isolated from the medicinal plants can be easily cultured and large-scale production of the drugs is possible through the fermentation process. It seems that using of plant cell extract in fungal artificial media is promising for the stable enhanced production of paclitaxel in fungi.

## Material and Methods

### Fungi and plant cell culture reagents

The medium components, plant growth regulators and paclitaxel standard used in this experiment were supplied by Sigma (USA) and Merck (Germany) Chemical Companies.

### Isolation of endophytic fungi from *T. baccata* and *C. avellana*

Healthy samples including the bark pieces, stem, bud and leaves were collected from *T. baccata* and *C. avellana* grown at the botanical garden of College of Agriculture and Natural Resources (36°40'01″N, 51°10'18″ E at an altitude of 1321 m), University of Tehran, located in Karaj, Alborz Province of Iran, in July and September 2014. The samples were treated with 75% ethanol (v/v) for 1 min and 2.5% sodium hypochlorite (w/v) for 2 min and rinsed two times with sterilized water. In order to test the effectiveness of surface sterilization^41^, 10 ml of the last rinsing water was centrifuged for 10 min at 10,000 g. The supernatant was removed and plated onto PDAC (PDA; supplemented with 250 mg/l Chloramphenicol). The surface sterilization was validated because no mycelial growth occurred. The surface disinfected small pieces (4 mm^2^) of inner bark, bud and leaf segments were excised and placed on the surface of PDAC in unique Petri dishes (100 × 15 mm), incubated at 25 °C to allow the growth of endophytic fungi. Pure fungal cultures of the endophytic isolates were prepared by the hyphal tip culture^42^. All fungal endophytes isolated from *T. baccata* and *C. avellana* were numbered as YEF# and HEF# series, respectively and stored on PDA at 4 °C.

### Culture, extraction and detection of paclitaxel

Two agar plugs (5 mm diameter) containing mycelia of the fungal isolates were cultured individually in 100 ml Erlenmeyer flasks containing 30 ml potato dextrose broth (PDB) medium. Cultures were incubated at 110 rpm at 25 °C for 12 days. The fungal mycelia were separated from the broth by filtration. This filtered culture was subsequently extracted by adding two volumes of dichloromethane^43^. The extracted solvent was evaporated using rotary evaporator (Eyela, Tokyo, Japan) to dryness at 35 °C. The dry residue was re-dissolved in 0.5 ml of absolute methanol. Intracellular paclitaxel was extracted from the mycelia with a procedure described below. Freeze-dried mycelia (100 mg) were soaked in 4 ml methanol, sonicated for 40 min. After centrifugation, the supernatant was removed and extracted with dichloromethane:water (1:1, v/v). The organic fraction was collected, dried under vacuum and resuspended in 0.5 ml methanol^44^. All samples were filtered through 0.22 µm cellulose acetate syringe filters before further analysis with high-performance liquid chromatography (HPLC) and high-performance liquid chromatography-mass spectrometry (LC-MS/MS).

Paclitaxel in samples was analyzed by the HPLC system (Waters, USA) with a C_18_ analysis column (MachereyeNagel EC 4.6 × 250 mm, 5 µm Nucleodur). The sample (20 µl) was injected each time and detected at 230 nm using a UV detector. The mobile phase was methanol:water (80:20 v/v) at a flow rate of 1.0 ml/min. The quantification of paclitaxel was based on an external standard of genuine paclitaxel (Sigma). Electrospray mass spectroscopy was done on fungal paclitaxel sample using the electrospray technique by an Alliance 2695 waters with a C_18_ analysis column (Eclipse Agilent 4.6 × 150 mm, 5 µm). The sample in 100% methanol was injected with a spray flow of 0.5 ml/min and a spray voltage of 4.5 kV by the loop injection method. The mobile phase was composed of acetonitrile acidified with 0.1% (v/v) formic acid (A) and water acidified with 0.1% (v/v) formic acid (B) with binary solvent-delivery gradient elution (0–5 min, 40–90% A, 60–10% B).

### Molecular studies: Genomic DNA extraction, PCR and sequencing

The endophytic fungus was cultured in PD broth at 25 °C with constant shaking for 7 days. The fungal mycelia were freeze-dried and the genomic DNA was extracted as described by Safaie *et al*.^45^. Briefly, 50 mg of fungal mycelia were vigorously crushed in liquid nitrogen to make a fine powder. The cells were lysed in 400 µl of DNA salt solution (Tris-HCl 100 mM, EDTA pH = 7.5–8 5 mM and NaCl 1.4 mM), mixed thoroughly and incubated at 65 °C for 15 min. The samples were cooled on crushed ice for 10 min and centrifuged at 13,000 g for 10 min at 4 °C. About 300 µl of the aqueous phase was transferred into a new labeled sterile tube. 210 µl cold isopropanol was added and mixed by inverting the tubes several times. The tubes were centrifuged for 15 min at 10,000 g and the supernatant was discarded, air dried and dissolved in 50 µl of sterile Millipore water.

The fungal internal transcribed spacer (ITS) fragments (ITS1-5.8S-ITS2) were amplified by PCR using the universal primers ITS1 and ITS4^46^. The actin gene (ACT) was partly amplified with primer pair ACT-512F and ACT-783R^47^ (Table S1). The PCR reaction mixtures (25 µl) consisted of 1 µl genomic DNA (~100 ng), 1 µl forward and reverse primers (10 pM), and 12.5 µl Premix Taq (TaKaRa Biotechnology Ltd., Japan), and 10.5 µl PCR quality water. The PCR reaction programs were an initial denaturation at 94 °C for 3 min, followed by 30 cycles of denaturation (94 °C for 30 s), annealing (56 °C (ITS) and (59 °C (ACT) for 30 s), extension (72 °C for 1 min) and a final extension at 72 °C for 5 min. The PCR products were analyzed by agarose gel electrophoresis and purified using a DNA gel extraction kit (Axygen Biotechnology Ltd., China). The purified PCR product was directly sequenced using the same primers by Bioneer (Shanghai, China).

The sequences of ITS1-5.8S-ITS2 region and actin gene of the endophytic fungus were compared with the data in National Center for Biotechnology Information, USA (NCBI) using BLAST search (http://blast.ncbi.nlm.nih.gov/Blast.cgi) to estimate the phylogenetic relationship. CLUSTAL X software (version 2.0, Conway Institute, USA) was used to generate the alignment of endophytic fungus^48^. Phylogenetic analysis was carried out by the neighbor-joining method using MEGA software (version 4.0, Biodesign Institute, USA). The bootstrap was 1,000 replications to assess the reliable level to the nods of the tree^49^.

### Preparation of cell extract (CE) and medium filtrate (MF)

The callus of hazel (*C. avellana*) was obtained from seed cotyledons on MS medium supplemented with 0.2 mg/l 6-benzylaminopurine and 2 mg/l 2,4-dichlorophenoxyacetic acid and solidified with 8 g/l agar agar^50^. The *C. avellana* CSC was established with transferring 5 g callus into 250 ml flasks containing 100 ml medium and were maintained at 25 °C in darkness on gyratory shakers at 110 rpm. Suspensions were also subcultured every 15 days until the cells reached homogeneity. Then 1.5 ± 0.1 g of hazel cells (fresh mass) was transferred to 100 ml flasks containing 30 ml of the cell culture medium. The cell culture medium and the culture conditions for growing cells of *C. avellana* remained the same as described above. Then the fresh cells were harvested on the 21^st^ day (the stationary growth phase)^50^ by passing CSC through a filter paper (Whatman No. 1). The cells washed several times with sterile double distilled water and dried at 60 °C. Then crushed thoroughly in liquid nitrogen. Crushed cells were soaked in water (100 mg/ml), sonicated for 20 min, mixed thoroughly and incubated at 65 °C for 30 min with continuous shaking. The hydrolysate centrifuged at 10,000 g for 15 min. After centrifugation, the supernatant (cell extract) was collected. The cell extract of hazel was divided into two parts: one part was autoclaved at 121 °C for 20 min and designated as autoclaved cell extract (ACE) and another part was filtered through 0.22 µm cellulose acetate syringe filters and designated as filter sterilized cell extract (FCE).

The spent medium was centrifuged at 12,000 g for 20 min to remove completely suspended cells. This cell-free medium was designated as medium filtrate. The medium filtrate of hazel CSC was divided into two parts: one part was autoclaved at 121 °C for 20 min and designated as autoclaved medium filtrate (AMF) and another part was filtered through 0.22 µm cellulose acetate syringe filters and designated as filter sterilized medium filtrate (FMF).

### Establishment of suspension culture of paclitaxel producing endophytic fungus

The experiment was carried out with three replications. Each replication consisted of 100 ml flask containing 30 ml MS medium supplemented with 0.2 mg/l BAP, 2 mg/l GA_3_ and 2 mg/l 2,4-D. The inoculum was prepared from 7-day-old cultures of strain YEF_2_ grown on PDA medium at 25 °C. Two mycelial agar plugs (5 mm diameter) per replication were cut from the margin of the growing colony using a sterilized cork borer. Cultures were maintained at 25 °C in darkness on gyratory shakers at 110 rpm.

Four plant elicitor preparations viz. ACE, FCE, AMF and FMF were added at different concentrations (7, 14, 28% (v/v)) to the medium at the time of culture. The control received an equal volume of the MS medium (for MF)/water (for CE). Since CSC of *C. avellana* produces paclitaxel^50^, elicitors derived from it may contain paclitaxel. As the control for fungal paclitaxel production, four elicitors derived from hazel CSC were added at different concentrations (7, 14, 28% (v/v)) to the culture medium with no inoculation and maintained in mentioned condition. The cultures were harvested on the 14^th^ day and analyzed for growth and paclitaxel production.

This experiment was designed as factorial based on a Completely Random Design (CRD) to determine how the CE and MF of *C. avellana* CSC affected the growth and paclitaxel production of paclitaxel-producing endophytic fungus strain YEF_2_. The factorial arrangement of the treatments was designed and consisted of three factors containing the type of elicitor with four levels, sterilization method with two levels and elicitor concentration with three levels, given 24 treatments.

### Statistical analysis

The hypothesis of normality and equal variance were met and conventional parametric statistics used for the analysis. Analysis of variance and means comparison using least significant difference (LSD) were performed by SAS (SAS 9.3, 2011) and SPSS (SPSS 15.0, 2006). Excel software (Excel, 2011) was used for making graphs. Simple linear regression of total paclitaxel against FW and DW for different treatments was developed by Statgraphics (54. Statgraphics Centurion XVII, 2015).

### Availability of data and material

The dataset supporting the conclusions of this article is included in the article.

## Electronic supplementary material

Supplementary Information
