# Supplementary material for: Elicitors Derived from Hazel (Corylus avellana L.) Cell Suspension Culture Enhance Growth and Paclitaxel Production of Epicoccum nigrum
Source: Sci Rep. 2018 Aug 13;8:12053. doi: 10.1038/s41598-018-29762-3 (PMC6089963; doi:10.1038/s41598-018-29762-3)
Supplement: Supplementary file 1 — Supplementary Information [file 41598_2018_29762_MOESM1_ESM.doc]

**Supplementary Materials**

**Elicitors Derived from Hazel (*Corylus avellana* L.) Cell suspension Culture Enhance Growth and Paclitaxel Production of *Epicoccum nigrum***

**Mina Salehi^1^, Ahmad Moieni^1^, Naser Safaie^2*^**

^1^ Plant Breeding and Biotechnology Department, Faculty of Agriculture, Tarbiat Modares University, Tehran, P.O. Box 14115-336, Iran

^2^ Plant Pathology Department, Faculty of Agriculture, Tarbiat Modares University, Tehran, P.O. Box 14115-336, Iran

^^Corresponding author: nsafaie@modares.ac.ir

1. **Figure Legends**

**Figure S1.** High-performance liquid chromatography (HPLC) spectra of standard paclitaxel (a) and paclitaxel extracted from fungal strain YEF_2_.

**Figure S2.** *Epicoccum nigrum* strain YEF_2_ grown in MS medium supplemented with 0.2 mg/L BAP, 2 mg/L 2,4-D, 2 mg/L GA_3_ and 3.5 % of filter sterilized cell extract (FCE) of *Corylus avellana* (a), 0.2 mg/L BAP, 2 mg/L 2,4-D and 3.5 % of FCE of *C. avellana* (b), 0.2 mg/L BAP, 2 mg/L 2,4-D and 2 mg/L GA_3_ (c) and 0.2 mg/L BAP, 2 mg/L 2,4-D (d).

**Figure S3.** *Epicoccum nigrum* strain YEF_2_ grown in MS medium supplemented with 0.2 mg/L BAP, 2 mg/L GA_3_, 2 mg/L 2,4-D and different concentration of filter sterilized cell extract of *Corylus avellana* (a to d, 7%, 5%, 3% and 0% (v/v), respectively).

**Figure S4.** *Epicoccum nigrum* strain YEF_2_ grown in PDB on the seventh day (a), on the 12^th^ day.


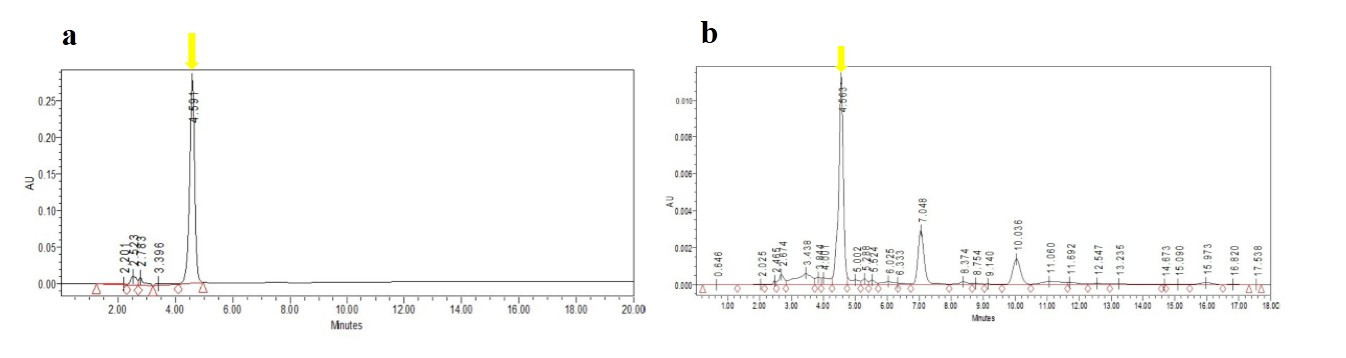


**Figure 1**


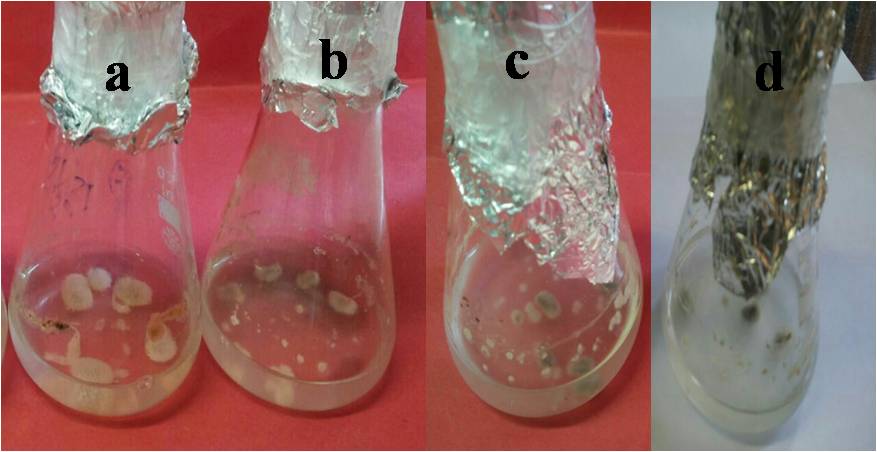


**Figure 2**


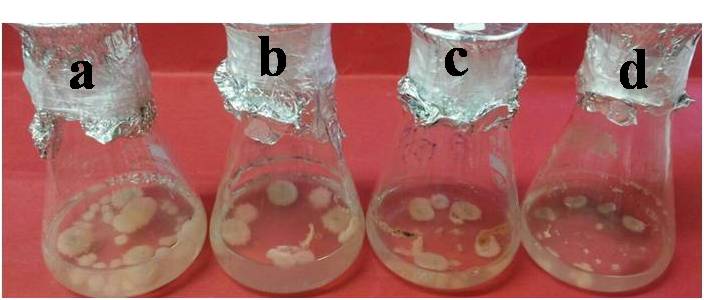


**Figure 3**


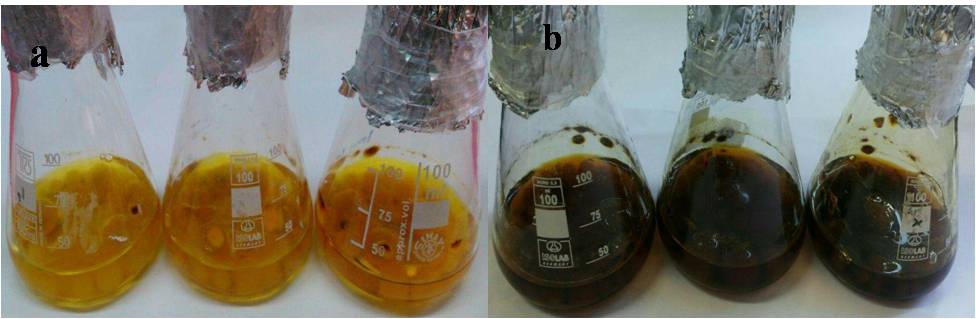


**Figure S4**

1. **Table Legends**

**Table S1.** Sequences of the primers used for the PCR analysis.

**Table S2.** Paclitaxel content (µg/L) of culture medium containing the elicitors derived from *Corylus avellana* cell suspension culture with no inoculation

| **Table S1**  Sequences of the primers used for the PCR analysis | | | |  |
| --- | --- | --- | --- | --- |
| Gene | Primers | Sequence (5′-3′ ) | PCR amplicon length | Reference |
| Actin | ACT-F | ATGTGCAAGGCCGGTTTCGC | 222 | Carbone and Kohn, 1999 |
|  | ACT-R | TACGAGTCCTTCTGGCCCAT |  |  |
| ITS1-5.8S-ITS2 rDNA | ITS1 | TCCGTAGGTGAACCTGCGG | 458 | White et al., 1990 |
|  | ITS4 | TCCTCCGCTTATTGATATGC |  |  |

| **Table S2**  Paclitaxel content (µg/L) of culture medium containing the elicitors derived from *Corylus avellana* cell suspension culture with no inoculation | | | | |
| --- | --- | --- | --- | --- |
| Treatment |  | Elicitor concentration (% (v/v)) | | |
|  |  | 7 | 14 | 28 |
| ACE |  | ND | ND | ND |
| FCE |  | 0.38 ± 0.032 | 0.82 ± 0.07 | 1.52 ± 0.28 |
| AMF |  | ND | ND | ND |
| FMF |  | 1.27 ± 0.14 | 2.28 ± 0.20 | 4.47 ± 0.32 |
| Abbreviations are as follows: ACE; Autoclaved cell extract, FCE; Filter sterilized cell extract, AMF; Autoclaved medium filtrate, FMF;Filter sterilized medium filtrate and ND, not detected. | | | | |
